# UK offshore immigration detention: why the medical community should act now

**DOI:** 10.1177/0141076821994271

**Published:** 2021-02-18

**Authors:** Ryan Essex, Erika Kalocsányiová, Frances Timberlake, Marta Welander

**Affiliations:** 1 Institute for Lifecourse Development, The 4918 University of Greenwich, Greenwich, London SE10 9LS, UK; 2 Refugee Rights Europe, London WC2H 9JQ, UK

In late September, reports emerged that the UK government had been drawing up plans to hold asylum seekers in detention centres overseas. Several news outlets reported on leaked government documents revealing ‘potential offshoring of asylum processing centres for those using clandestine entry routes to the UK’.^1^ Locations under consideration include British Overseas Territories as well as Moldova, Morocco and Papua New Guinea. At this point, the Home Office has given little away; however, sources have suggested that the government is presently looking at ‘every option that can stop small boat crossings and fix the asylum system’,^2^ while other reports suggest that the Home Office has already carried out assessments for an offshore centre on Ascension Island, over 4000 miles from the UK.^2^

The UK’s policies of ‘*non-entrée*’ are of course nothing new – it has a long history of seeking to prevent the arrival of asylum seekers, not least through its extraterritorial ‘juxtaposed controls’. With immigration checks taking place prior to passengers boarding a train or ferry rather than upon arrival in the UK, the UK border has in practice been moved from Dover to seven locations in Belgium and France (Calais, Calais-Fréthun, Dunkirk, Coquelles, Paris, Brussels and Lille). The juxtaposed arrangements have been heavily criticised by rights groups arguing that this policy, in the absence of means to access the UK asylum system, contributes to a breach of the UK’s international legal obligations by ‘circumventing the right to asylum, and as a result, also the protection against *non-refoulement*’^3^ (also see: https://refugee-rights.eu/wp-content/uploads/2020/11/RRE_PP_NewWaysAccessUKAsylum-2020.pdf). In addition to these controls, within the UK the government has continued to defend its policy of indefinite detention despite being the only European country to have no statutory time limit. The UK’s most recent plans, to hold asylum seekers in offshore detention centres would take matters to an entirely new level, raising a range of additional concerns.

There are already many well-founded reasons to oppose the dangers of detention of asylum seekers. When operated offshore, these dangers only increase, and thus opposition is vital; on human rights grounds, for the lack of transparency and accountability that results, for financial and logistical reasons, or because of the simple fact that the UK has the capacity to help. There are also well-founded reasons to oppose offshore detention on health grounds, with a substantial evidence base that comes from the Australian experience of offshore detention on which the UK appears to be modelling itself.

While relatively few people seek asylum in Australia, over the last three decades successive Australian governments have implemented increasingly harsh measures aimed at deterring asylum seekers, especially those travelling to Australia by boat. Throughout the Asia-Pacific region, Australia has invested heavily in policies and infrastructure aimed at immobilising asylum seekers and for decades has even turned asylum seeker boats back at sea.^4^ Arguably the most controversial of these deterrence measures, however, has been the use of offshore immigration detention. Australia first established offshore detention centres on Manus Island (Papua New Guinea) and Nauru, from 2001 to 2008. This policy was more recently reintroduced, and since 2013 boats with asylum seekers bound for Australia have again been sent to Nauru and Papua New Guinea, this time with no chance of resettlement in Australia. Thousands were detained offshore for a number of years and seven years later hundreds still await news about possible third-country resettlement. Investigation and testimony have shed light on riots, physical and sexual abuse (of adults and children) and violence, issues which have persisted for over seven years.^5^ Australia’s offshore asylum policies have been called ‘cruel, inhuman, or degrading treatment’ by the International Criminal Court^6^ and ‘state-sanctioned child abuse’ by the Australian Medical Association.^7^ Amnesty International recently concluded that, ‘The conditions on Nauru – refugees’ severe mental anguish, the intentional nature of the system, and the fact that the goal of offshore processing is to intimidate or coerce people to achieve a specific outcome – amounts to torture’.^8^ Rather than act on these issues and take steps toward a more humane approach, the Australian government has instead dismissed such concerns and attacked critics, insisting that this approach is necessary as a means of deterring others that would otherwise seek Australia’s protection.

Australian healthcare professionals have been central to the day-to-day functioning of Australian immigration detention centres and also instrumental in bringing to light the devastating impacts of offshore detention, as well as in opposing the country’s offshore asylum policies. Much has been written about healthcare within Australian immigration detention centres, and arguably few contemporary issues have been as vexing for the healthcare community. At the heart of these issues remains the fact that immigration detention is antithetical to health and wellbeing; it violates almost every human rights instrument to which Australia is signatory and is an affront to the dignity of those who are detained.

While the government has long blocked researchers from accessing detention centres, some recent studies begin to quantify the harms to health in more detail. Médecins Sans Frontières’ (MSF) ‘Indefinite Despair’ report, for example, shows that among the 208 refugee and asylum seekers assessed by MSF on Nauru, 62% were diagnosed with moderate to severe depression, 25% with an anxiety disorder and 18% with PTSD, among a range of other psychiatric diagnoses.^9^ For the 74 refugees and asylum seekers seen over time, 15 (20%) remained stable, while 51 (69%) deteriorated and only 8 (11%) showed improvement in their daily functioning. More recently, Hedrick et al.^10^ utilised health records to analyse episodes of self-harm between August 2014 and July 2015. Rates of self-harm were found to be 260 per 1000 asylum seekers on Nauru, meaning rates of self-harm in offshore detention were up to 216 times higher than that seen in the Australian community. Beyond the mental health impacts, offshore immigration detention has also resulted in numerous deficits in the delivery of healthcare. One of the most pressing issues related to offshore detention has been the transfer of those who are unwell to the Australian mainland. That is, the Australian government has long resisted transferring people to Australia for medical treatment, with the government refusing to move suicidal children to the Australian mainland. On a number of occasions this has had fatal consequences, with multiple deaths reported from issues that would have otherwise been preventable.^11^

There are lessons that can be taken from the Australian healthcare community in its opposition to offshore detention as well. Recognising that the Australian government has been unmoved by evidence and the harms of these policies, healthcare professionals have been instrumental in bringing to light the devastating health impacts of offshore detention, whistleblowing and even engaging in acts of civil disobedience.^5^ Such evidence and action have been relatively successful in Australia. While offshore detention remains, a number of small victories can be counted. Children are now no longer detained onshore or on Nauru,^12^ and many people who needed urgent medical intervention offshore have now been transferred to Australia for treatment.^13^ In 2018, for example, amid increasingly disturbing reports about the health of detainees offshore, the Australian government passed what became known as the Medevac legislation, a law which strengthened doctors’ positions to advocate for those offshore to be transferred. While this legislation was repealed in late 2019, the healthcare community was instrumental in pushing for its introduction and in resisting its repeal, coordinating with lawyers and placing pressure on the government. During the time Medevac was in force, hundreds of unwell refugees were transferred to the mainland.^11^

Beyond the Australian experience, there are already a number of warning signs closer to home, with the British Medical Association raising concerns about the current immigration detention policies in the UK.^14^ Likewise, medical organisations including Doctors of the World and Freedom from Torture (https://www.doctorsoftheworld.org.uk/wp-content/uploads/2020/11/Letter-on-the-use-of-MoD-sites-as-accommodation_26.11.2020.pdf) have already warned of the health impacts of the detention-like conditions in the military barracks already being used to house asylum seekers. Further warning can be found in the UK’s current policies, with widely unscrutinised use of UK detention facilities on French soil.^15^ While these short-term holding facilities have a 24-hour time limit and are intended only to hold people with incorrect documentation at the border controls in Calais and Dunkirk, the lack of oversight and accountability due to ‘lack of jurisdictional clarity’, their poor access to healthcare and their relative invisibility, provide an exemplar of the issues that would be encountered in more comprehensive UK detention offshoring. Offshore detention would only exacerbate these issues and Australia’s approach should serve as a warning to the UK government and healthcare community alike. While the UK is looking to other countries such as Australia for ‘solutions’, they also need to look at the consequences of these policies, with offshore detention having a devastating impact on health and wellbeing. The healthcare community should and could take a stand against these policies, which are at best antithetical to health and at worst a human rights disaster in the making.

## References

[1] Pegg D and Lewis P. Australian-style offshore asylum plan driven by No 10. *The Guardian*. See https://www.theguardian.com/uk-news/2020/sep/30/australian-style-offshore-asylum-plan-driven-by-no-10 (last checked 3 February 2021).

[2] BBC News. *Ascension Island: Priti Patel Considered Outpost for UK Asylum Centre Location*. See https://www.bbc.co.uk/news/uk-politics-54349796 (last checked 3 February 2021).

[3] Amnesty International. *The Human Rights Risks of External Migration Policies*. See https://www.amnesty.org/en/documents/pol30/6200/2017/en/ (last checked 3 February 2021).

[4] Nethery A Rafferty-Brown B Taylor S. Exporting detention: Australia-funded immigration detention in Indonesia. J Refugee Stud 2013; 26: 88–109.

[5] Essex R. The Healthcare Community and Australian Immigration Detention: The Case for Non-Violent Resistance, London: Palgrave Macmillan, 2020.

[6] Doherty B. Australia’s offshore detention is unlawful, says international criminal court prosecutor. *The Guardian*. See https://www.theguardian.com/australia-news/2020/feb/15/australias-offshore-detention-is-unlawful-says-international-criminal-court-prosecutor (last checked 3 February 2021).

[7] Owler B. *Speech to AMA Forum on Health of Asylum Seekers 2016.* See https://ama.com.au/media/ama-speech-prof-owler-ama-asylum-seeker-health-forum (last checked 3 February 2021).

[8] Amnesty International. Island of Despair: Australia's “processing” of Refugees on Nauru, London: Amnesty International, 2016.

[9] Médecins Sans Frontières. *Indefinite Despair: The Tragic Mental Health Consequences of Offshore Processing on Nauru*. See https://msf.org.au/article/statements-opinion/indefinite-despair-mental-health-consequences-nauru (last checked 3 February 2021).

[10] Hedrick K Armstrong G Coffey G Borschmann R. Self-harm in the Australian asylum seeker population: a national records-based study. SSM-Population Health 2019; 8: 100452–100452. 31440577 10.1016/j.ssmph.2019.100452 PMC6698923

[11] Talbot A Newhouse G. Strategic litigation, offshore detention and the Medevac Bill. Court Consci 2019; 13: 85–85.

[12] ABC News. Last remaining asylum seeker children on Nauru to leave the island for the US, Scott Morrison confirms. *ABC News*. See https://www.abc.net.au/news/2019-02-03/nauru-last-asylum-seeker-children-to-leave-detention-pm-says/10774910 (last checked 3 February 2021).

[13] Royal Australian College of Physicians. *Over 5,000 Doctors Sign Open Letter to Save Medevac Ahead of Possible Senate Vote*. See https://www.racp.edu.au/news-and-events/media-releases/over-5-000-doctors-sign-open-letter-to-save-medevac-ahead-of-possible-senate-vote (last checked 3 February 2021).

[14] British Medical Association. *Locked Up, Locked Out: Health and Human Rights in Immigration Detention*. See https://www.bma.org.uk/media/1862/bma-locked-up-locked-out-immigration-detention-report-2017.pdf (last checked 3 February 2021).

[15] Bosworth M. Immigration detention and juxtaposed border controls on the French north coast. *Eur J Criminol.* EPub ahead of print 7 February 2020. DOI: 10.1177/1477370820902971.

